# Cognitive Representation of Human Action: Theory, Applications, and Perspectives

**DOI:** 10.3389/fpubh.2016.00024

**Published:** 2016-02-18

**Authors:** Christian Seegelke, Thomas Schack

**Affiliations:** ^1^Neurocognition and Action Research Group, Faculty of Psychology and Sport Sciences, Bielefeld University, Bielefeld, Germany; ^2^Center of Excellence Cognitive Interaction Technology (CITEC), Bielefeld, Germany; ^3^Research Institute for Cognition and Robotics (CorLab), Bielefeld, Germany

**Keywords:** cognitive representation, motor control, memory, action planning, applied research, technology

## Abstract

In this perspective article, we propose a cognitive architecture model of human action that stresses the importance of cognitive representations stored in long-term memory as reference structures underlying and guiding voluntary motor performance. We introduce an experimental approach to ascertain cognitive representation structures and provide evidence from a variety of different studies, ranging from basic research in manual action to application-oriented research, such as athlete performance and rehabilitation. As results from these studies strongly support the presence of functional links between cognitive and motor processes, we regard this approach as a suitable and valuable tool for a variety of different disciplines related to cognition and movement. We conclude this article by highlighting current advances in ongoing research projects aimed at improving interaction capabilities in technical systems, particularly for rehabilitation and everyday support of the elderly, and outline future research directions.

## Introduction

Motor activities within particular environmental conditions are central dimensions of biological organism since millions of years. Important stages in evolution are mainly based on the establishment of new functional links between the motor system, related memory structures, and the perception of biological systems. Furthermore, motor actions – such as dance or sports – have always been an important element in all human cultures. Stated in a more dramatic language: “from the motor chauvinist’s point of view the entire purpose of the human brain is to produce movement … [and] all sensory and cognitive processes may be viewed as inputs that determine future motor outputs” [Ref. (1), p. 487]. Consequently, understanding how we plan and control our bodily actions (i.e., the topic of motor control research) is not only of theoretical importance but also has large and diverse practical relevance.

For example, research in motor control contributes at exploring the principles underlying elite performance of professional athletes and musicians and devising training appropriately (2–5). It can also aid clinical practice by consulting physicians, occupational therapists, and physiotherapists in terms of development and implementation of (neurocognitive) motor rehabilitation treatments for people suffering from motor disorders (6–9). More generally, motor control research can therefore contribute to a more independent and self-determined everyday life from childhood to the elderly. Furthermore, it can help engineers and roboticists in developing technical systems and prosthetic devices that dispose more “human-like” action capabilities, and hence, are intuitive and easy to operate for humans users (10–12).

In this article, we take a perceptual–cognitive perspective to motor control emphasizing the strong functional connections between cognitive and motor processes underlying action control. From our point of view, human motor actions are not isolated events with defined start- and endpoints but are built upon evolved hierarchical structures consisting of different levels and modules.

## Cognitive Representation of Action: A Theoretical Framework

In planning a movement, the brain must select one of the many possible movements. Known as the degrees of freedom problem (13), it acknowledges the fact that due to the redundant anatomical, kinematic, and neurophysiological degrees of freedom in the motor system, there are multiple ways in which a movement can be performed to achieve the same action goal. Consequently, motor control can be considered “the process of mastering the redundant degrees of freedom of the moving organ … its conversion to a controllable system” [Ref. (13), p. 127].

Although the subject of motor control has long been a topic of interest primarily for the neurosciences (14), in the past few years, a growing interest in this topic emerged in the fields of cognitive science and psychology. Current theoretical conceptions in cognitive psychology on action control share the belief that actions are guided by internally represented action goals and their anticipated (perceptual) features [e.g., Ref. (15–19)]. Interestingly, these perspectives are reminiscent of earlier ideas of Bernstein (13) regarding the construction of movement. Bernstein explicitly emphasized the importance of sensory feedback processing and anticipation in realizing any type of goal-directed motor act, and that any voluntary motor action cannot be initiated without a model of what should result from the planned action. This idea is reflected in his model of the desired future (i.e., a model of what should be), which is supposed to play an important role in controlling motor acts. Such a model must possess the capability to form a representation of future events by integrating information from past (i.e., memory) and present (i.e., sensory) events in order to generate motor commands that transform the current state in the sensory environment into the desired state (i.e., achieving the action goal).

**Figure 1:**
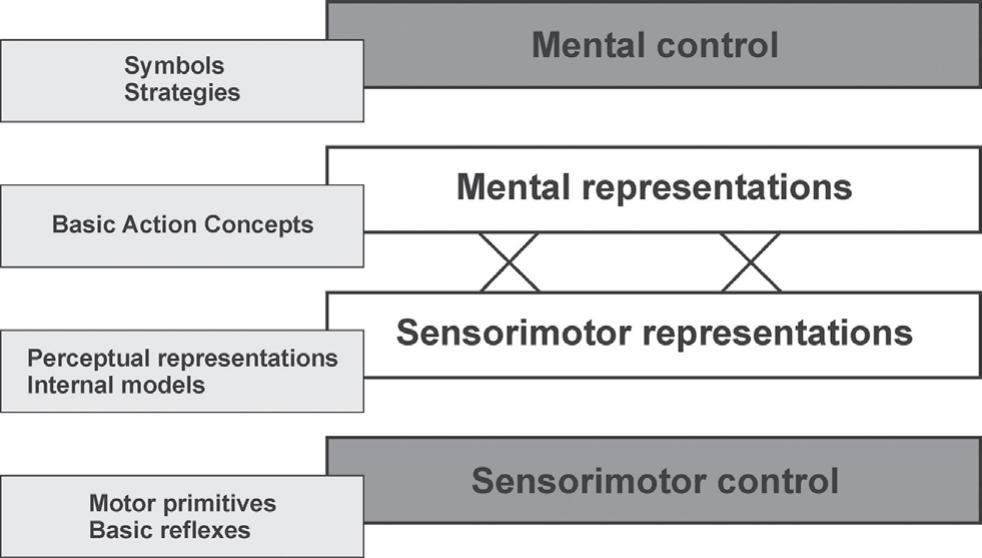
**Cognitive architecture (levels) of motor action and corresponding tools [modified from Ref. (20, 24)]**.

Expanding this idea, we have proposed a cognitive architecture model, which views the functional construction of actions on the basis of a reciprocal assignment of performance-oriented regulation levels and representational levels [Ref. (11, 20–22); see Figure 1]. According to this view, basic action concepts (BACs), stored hierarchically in long-term memory (LTM), are thought to serve as major representation units for movement control. Analogous to the well-established notion of basic concepts for objects (23), BACs are considered the mental counterparts of functionally relevant elementary components or transitional states (body postures) of movements. BACs are based on the cognitive chunking of body postures and movement events concerning common functions in realizing action goals. In contrast to basic object concepts, they do not refer to behavior-related invariance properties of objects but to perception-linked invariance properties of movements. Consequently, BACs can be understood as representational units in memory that tie together the functional and sensory features of movements. The integration of sensory features refers to the perceptual movement effects, whereas the functional features are derived from the action goals. Taken together, such movement representations provide the basis for action anticipation and control by linking higher level action goals with the lower-level perceptual effects in the form of cognitive reference structures.

## Measuring Cognitive Representations

**Figure 2:**
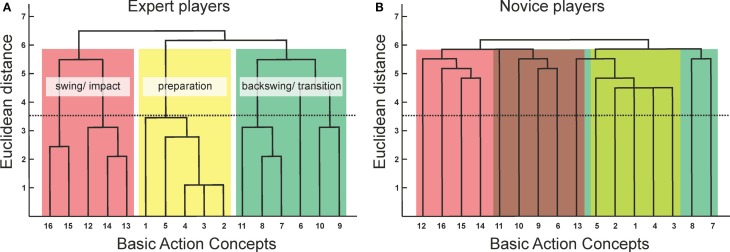
**Dendrograms for expert players (A) and novice players (B) based on the hierarchical cluster analysis of the golf swing**. The numbers on the horizontal axes relate to the basic action concepts (BACs). The numbers on the vertical axes display Euclidean distances. The lower the Euclidean distance between two concepts in feature space, the stronger the link between these concepts. The horizontal dotted line marks the critical distance *d*_crit_ for a given alpha-level (*d*_crit_ = 3.46, *p* = 0.05): links below this line are considered as statistically relevant. BACs are as follows: (1) position club face, (2) grip control, (3) address position, (4) ball position, (5) locking, (6) push club away, (7) pressure inside right foot, (8) bending right knee, (9) arms make wide circle, (10) cock the wrists, (11) back points to target, (12) left side out of the way, (13) head behind the ball, (14) acceleration through the ball, (15) let go, and (16) balance at finish. The experts’ cluster solutions reflect the functional movement phases of the golf swing: preparation (BAC 1–5), backswing and transition (BAC 6–11), and downswing and impact (BAC 12–16), whereas novices’ dendrograms do not exhibit any statistically relevant cluster solutions.

A particularly promising method to assess structures of cognitive representation in LTM constitutes the so-called structure dimensional analysis-motorics [SDA-M; (20, 25)]. The SDA-M procedure ascertains relational structures in a given set of concepts. The internal grouping of conceptual units (i.e., the clustering of BACs) delineates the structure of the cognitive representation of a certain movement. Whereas most of the methods aimed at assessing knowledge-based cognitive representations of movements in LTM focus on explicit knowledge [e.g., interviews, questionnaires; see Ref. (26) for a review], an important advantage of the SDA-M is that it allows for a psychometric analysis of the structures without necessitating participants to give explicit statements regarding their representation, but rather through means of knowledge-based decisions in an experimental setting. The SDA-M consists of four steps [for further details, see Ref. (25)]: first, a splitting procedure provides an Euclidean distance scaling between BACs of an appropriate predetermined set. Specifically, participants are required to subjectively decide whether or not a given BAC is functionally related to another BAC (i.e., pair-wise comparison). During this process, a randomly selected BAC from a predetermined set is presented as reference item (or anchor), and all other BACs of the set are successively compared to the anchor item. Participants have to decide whether or not the two given concepts are functionally related to each other during movement execution. Through this procedure, the list of BACs is split into two subsets, a positive (i.e., functionally related) and a negative (i.e., functionally not related) subset, and this procedure is repeated until each BAC was once in the anchoring position and compared to all other BACs. Based on these decisions, the positive and negative subsets are summed separately, providing an Euclidean distance scaling between the BACs. Second, a hierarchical cluster analysis transforms the set of BACs into a hierarchical structure (i.e., a dendrogram; Figure 2). Third, a dimensioning of the cluster solutions through a factor analysis is performed, resulting in a factor matrix classified by clusters. Finally, a within- and between-group comparison of the cluster solutions is used to determine their structural invariance.

As we will outline in the next section, the SDA-M has been utilized, either alone or in conjunction with other experimental techniques, in a variety of different studies, thereby encompassing basic research in manual action as well as more applied research in the context of athlete performance, rehabilitation, and cognitive robotics (11, 12, 21, 22). As such the SDA-M provides an effective means by which functional relationships between cognitive representations and motor performance can be assessed, making it a valuable tool for scientists in basic and applied research, as well as practitioners working with athletes or patients.

## Empirical Evidence and Applications

One way to ascertain links between motor performance and cognitive representation structures is to examine differences between groups of different motor expertise. Schack and Mechsner (27) took this approach and compared the cognitive representation of the tennis serve in expert players, amateur players, and novices. Based on ratings given by tennis experts and coaches, the authors defined 11 BACs in relation to the functional movement structure derived from biomechanical movement parameters. The results of this study showed that expert players exhibited representation structures that had a distinct hierarchical organization, were remarkably similar between individuals, and reflected the three functional phases (i.e., pre-activation, strike, and final swing) of the movement. By contrast, novices’ representation structures were organized less hierarchically, exhibited greater variability between individuals, and did not match the functional task demands. Similar systematic relationships between cognitive representation structures and expertise have been reported in a number of sport contexts, such as dancing, judo, windsurfing, soccer, volleyball, and gymnastics (24, 28–32).

Differences in the cognitive representation structure depending on the level of expertise suggest that improvements in motor performance involve changes in the corresponding representation structure. Accordingly, motor learning can be regarded as the modification of representation structures in LTM (12, 20). Frank et al. (32) directly addressed this assertion by examining the effects of movement practice on the representation structure during early skill acquisition of a gold putt, using a pre–posttest design. Novice golfers were randomly assigned to a practice or control group, and participants in the practice group performed a total of 600 golf putts over the course of three training days. Results indicated that along with improvements in putting performance, there were significant changes within the practice group’s cognitive representation (i.e., it became more similar to an expert structure), suggesting that motor learning is linked to functional adaptations in the cognitive representation structure.

As the cognitive representation structures of complex skills can be analyzed not only on a group level but also on an individual level (29, 33), the SDA-M can be used as a diagnosing tool to derive knowledge about an athlete’s individual skill representation. This information can then be utilized by coaches and athletes to identify specific movement problems, and hence, can build the basis for further practical work in coaching, technical preparation, and mental training (24, 30).

We would like to emphasize, however, that the usefulness of this approach is by far not limited to complex actions (such as in sports) but has a much broader spectrum of application such as for actions required in everyday life, such as walking (34) or object manipulation (35). Thus, this approach might be also a valuable source for people interested in health-related issues. To exemplify our point, consider the following: an important component of (mental) health is that we acquire, maintain, and rebuild (after loss) independence in everyday life (36). Many everyday activities require that we grasp and manipulate objects (e.g., cooking, cleaning, and getting dressed). Although we typically pay very little attention to how we accomplish such tasks, it is apparent that the inability to perform these actions (be it acquired or congenital) has dramatic consequences for our everyday life. Consequently, we argue that it is important to understand how such manual actions often required in everyday activities are controlled and represented, for example, to diagnose certain motor problems and to develop suitable intervention in order to maintain or rebuild a certain level of independence.

In this regard, the SDA-M has already been proven a promising tool. Specifically, Stöckel et al. (35) examined links between anticipatory motor planning in manual action and the development of cognitive representations of grasp postures in children aged 7–9 years. Motor planning skills were assessed *via* the so-called bar-transport task (37). In this task, participants are required to grasp a horizontally oriented bar (using either an overhand or an underhand grip) and place it with either its left or right end on a target. It is typically found that neurologically healthy adults select initial grasp postures that will result in comfortable thumb-up postures when placing the bar on the target. Termed the end-state comfort effect, this finding supports the notion that people represent future body postures and plan initial grasp postures in anticipation of the future states [see Ref. (38, 39) for reviews]. Cognitive representations of grasp postures were assessed *via* the SDA-M with pictures of a hand grasping common objects (e.g., hammer, scissors, and glass) as BACs. In line with other studies on motor planning during childhood [see Ref. (40) for a review], Stöckel et al. (35) found that end-state comfort satisfaction increased with age, and the 9-year-old children had more distinct representation structures of grasp postures than the 7- and 8-year-old children. Importantly, the sensitivity toward comfortable end-postures was related to the cognitive representation structure. Children who exhibited grasp comfort-related and functionally well-structured representations also showed a stronger preference for end-state comfort in the bar-transport task, supporting the notion that cognitive action representation plays an important role in the planning and control of grasp postures.

Of particularly practical relevance for our argument is a study conducted by Braun et al. (41) in the context of rehabilitation. Specifically, the authors examined the cognitive representation of a common everyday activity – drinking from a cup – in elderly patients recovering from stroke and matched controls. Although the representation structures of the controls reflected the functional action phases and were very similar across participants, the patients’ structures differed largely from each other and hardly featured any functional structure. Thus, this study demonstrates that the SDA-M can also be used as a diagnostic tool for therapists in clinical and rehabilitation contexts (34).

## Current Advances and Perspectives

Although the studies presented above clearly demonstrate that the SDA-M is a viable and versatile tool that can be employed in variety of different contexts, it has not, as of yet, exploited its full potential, especially in clinical settings. Just to name one example, it could be applied to children suffering from developmental coordination disorder (DCD). These children have difficulties in learning new motor skills, are not able to predict the outcome of their movement, and do not easily recognize movement errors (42, 43), which affects their performance in the classroom and activities of daily living (44). As this deficit, according to our view, is likely to be related to non-functional cognitive action representations, the SDA-M could be used to support other commonly used intervention techniques (45) to improve children’s day-to-day activities.

A similar goal is pursued by the current research project adaptive cognitive training (ACT) in which our research group collaborates with a local non-profit making foundation, in which job-related knowledge (e.g., serving and cooking) is transmitted to mentally handicapped people. By assessing the cognitive representations of such job-related activities in these individuals and providing individualized feedback, a central aim of ACT is to stimulate the developmental potential of handicapped people to foster their integration into normal working and daily routines.

In light of the demographic change, facilitating and maintaining independence in daily activities, particularly for the elderly, are also central objectives of two other ongoing research projects – adaptive and mobile action assistance in daily living activities (ADAMAAS) and KogniHome – in which our group collaborates with several partners from science and industry. Central and common to these projects is that they utilize, integrate, and advance interaction capabilities of state-of-the-art technologies in order to assist people in everyday activities. ADAMAAS focuses on the development of a mobile adaptive assistance system in the form of intelligent glasses that provide unobtrusive and intuitive support in everyday situations (e.g., baking, making coffee, repairing a bike, etc.). It is intended that the system will identify problems in ongoing action processes, react to errors, and provide context-related assistance in textual, pictorial, or avatar-based formats superimposed on a transparent virtual display. The project integrates cognitive representation analysis, eye tracking, physiological measures (pulse, heart rate), computer vision (object and action recognition), and augmented reality with modern diagnostic and corrective intervention techniques. The uniqueness of this system is its ability to react to errors in real-time, provide individualized feedback for action support, and learn from expert models as well as the individual behavior of the user.

To sum up, we put forward a cognitive perspective to action control that stresses the importance of cognitive representations stored in LTM as reference structures underlying and guiding voluntary motor performance. We introduced an experimental method (the SDA-M) used to ascertain cognitive representation structures and provided evidence from both basic and applied research that reinforce the proposition of functional links between cognitive and motor processes. Thus, we view this approach as a viable and versatile tool, capable of providing individualized recommendations across a range of different contexts. Alongside and in combination with the ongoing advances in developing, improving, and integrating interaction capabilities in technical systems, this perspective constitutes a promising route in order to acquire, maintain, and rebuild independence in everyday life activities across human development, and thus, contribute to public health.

## Author Contributions

CS and TS contributed to the conception of the present work. CS drafted the first version of the manuscript and TS helped in revising it. All authors approved the final, submitted version of the manuscript.

## Conflict of Interest Statement

The authors declare that the research was conducted in the absence of any commercial or financial relationships that could be construed as a potential conflict of interest.
